# Daily quality assurance phantom for ultrasound image guided radiation therapy

**DOI:** 10.1120/jacmp.v8i3.2467

**Published:** 2007-06-29

**Authors:** Laura Drever, Michelle Hilts

**Affiliations:** ^1^ Department of Medical Physics BC Cancer Agency Victoria British Columbia Canada

**Keywords:** Ultrasound imaging, phantoms, quality assurance

## Abstract

A simple phantom was designed, constructed, tested, and clinically implemented for daily quality assurance (QA) of an ultrasound‐image‐guided radiation therapy (US‐IGRT) system, the Restitu Ultrasound system (Resonant Medical, Montreal, QC). The phantom consists of a high signal echogenic background gel surrounding a low signal hypoechoic egg‐shaped target. Daily QA checks involve ultrasound imaging of the phantom and segmenting of the embedded target using the automated tools available on the US‐IGRT system. This process serves to confirm system hardware and software functions and, in particular, accurate determination of the target position. Experiments were conducted to test the stability of the phantom at room temperature, its tissue‐mimicking properties, the reproducibility of target position measurements, and the usefulness of the phantom as a daily QA device. The phantom proved stable at room temperature, exhibited no evidence of bacterial or fungal invasion in 9 months, and showed limited desiccation (resulting in a monthly reduction in ultrasound‐measured volume of approximately 0.2 cm^3^). Furthermore, the phantom was shown to be nearly tissue‐mimicking, with speed of sound in the phantom estimated to be 0.8% higher than that assumed by the scanner calibration. The phantom performs well in a clinical setting, owing to its light weight and ease of operation. It provides reproducible measures of target position even with multiple users. At our center, the phantom is being used for daily QA of the US‐IGRT system with clinically acceptable tolerances of ±1 cm^3^ on target volume and ±2 mm on target position. For routine daily QA, this phantom is a good alternative to the manufacturer‐supplied calibration phantom, and we recommended that that larger phantom be reserved for less frequent, more detailed QA checks and system calibration.

PACS numbers: 87.66.Xa, 87.63.Df

## I. INTRODUCTION

Modern three‐dimensional conformal radiation therapy and intensity‐modulated radiation therapy improve dose conformity around planning target volumes (PTVs), reducing the dose to adjacent normal structures. The opportunity to fully exploit these treatment techniques by reducing PTV margins is enticing, especially considering the possibility for dose escalation and the ensuing potential for increased tumor control that reduced margins may allow.^(^ ^1^ ^,^ ^2^ ^)^ However, with the reduction of PTV margins comes an increased probability of geometric miss resulting from setup error and organ motion. This risk can be reduced or avoided with implementation of accurate image‐guided radiation therapy (IGRT) to localize the target daily at time of treatment.

Ultrasound (US) is one method of performing IGRT for prostate cancer, and several devices are commercially available for this purpose. Most US‐IGRT systems operate by comparing US images obtained at time of treatment to X‐ray computed tomography (CT) images obtained at time of planning so as to measure daily prostate misalignments. However, this “cross‐modality” comparison approach has inherent difficulties (in part because the prostate base is frequently difficult to visualize on CT), and discrepancies between US and other IGRT approaches have been reported.^(^ ^3^ ^–^ ^6^ ^)^

A relatively new system, Restitu (Resonant Medical, Montreal, QC), offers an alternative by incorporating an US system in the CT simulation room in addition to the US system in the treatment room. This second US system is used to acquire an US reference scan at the time of planning and allows for an intramodality comparison of planned and treatment images. In both rooms, an infrared imaging system that tracks the position of the US probe is used to relate the US scans to the room coordinates and to the machine isocenter. This ceiling‐mounted camera system is located at the foot of each treatment couch. Recent results indicate that this intramodality approach provides more accurate measures of prostate misalignment than does the conventional cross‐modality approach.^(7)^

For accurate operation of the US‐IGRT system, the US installations in the CT and treatment rooms must be calibrated to match the relevant room coordinate systems. (In addition, the CT and LINAC coordinate systems must correspond, as for any CT planning–based radiation therapy). A calibration and quality assurance (QA) phantom supplied by the manufacturer can be used effectively to test system accuracy in each room, to perform system calibration, and to test the integrity of the system across the CT and treatment rooms. We currently perform regular weekly and monthly QA checks on the US‐IGRT system using this calibration phantom. However, we are reluctant to use this phantom for the routine daily QA checks that are required in the treatment room. Because the calibration phantom is critical for system calibration (and therefore system operation), we believe that it should be reserved for that purpose and for detailed weekly and monthly QA procedures. Furthermore, the calibration phantom is quite heavy and awkward to move, and could easily be dropped. The purpose of the present work was to design, manufacture, test, and clinically implement a phantom for routine daily QA of an US‐IGRT system.

The daily QA phantom would be used in the daily testing that determines whether the US‐IGRT system in the treatment room is correctly calibrated with the treatment and CT room coordinate systems. Correct system calibration is essential for accurately matching patient treatment position to the planned position determined at CT simulation. The calibration check should be accomplished by aligning the phantom with the treatment room lasers, acquiring an US image set, using the provided software tools to segment the images, and then having the system compute the target volume and the centroid position. By comparing the values obtained with baseline values determined at the time of system calibration, any errors in system performance can be detected—permitting a re‐calibration to be performed, if required.

## II. MATERIALS AND METHODS

### A. Phantom design and manufacture

Several characteristics required for the daily QA phantom limited the materials that could be used in its construction and guided the design process. To provide accurate spatial mapping, the speed of sound in the phantom material should be similar to the average speed of sound in human tissue assumed by the US scanner. However, because the phantom would be used for relative daily checks and not for absolute measurements or system calibration, this requirement, although desirable, could be somewhat relaxed. Second, to permit testing of the segmentation algorithm employed by the US‐IGRT system, the phantom should provide high‐quality images showing an easily identifiable target. A further requirement was that the phantom should be relatively stable during storage at room temperature. Finally, as a routine daily use QA device, the phantom should be relatively small and lightweight (on the order of a kilogram).

A review of the literature suggests that ultrasound phantoms can be made of several gelling agents such as food‐grade gelatin^(8)^ and high‐strength agarose gel(^9)^. To limit costs, we used a porcine gelatin to build an initial prototype; however, that phantom did not meet the requirement of stability at room temperature, and so high‐strength agarose gel was used in the final model.

**Figure 1:**
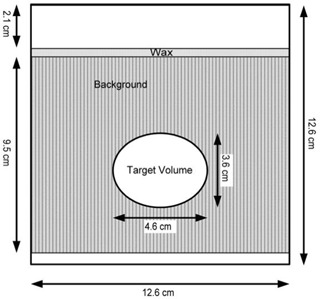
Schematic of the phantom, showing the target and the background.

The phantom design consists of a high‐signal echogenic background surrounding a low‐signal hypoechoic egg‐shaped target (Fig. 1). The background and target are both composed of a high‐strength agarose gel (1.5% by weight: Sigma–Aldrich, St. Louis, MO) with potassium sorbate (0.2% by weight) added to control growth of bacteria and fungi. The gel was made using regular tap water. The high‐signal background was created by adding 20.8 g/L finely powdered graphite [diameter: 44 μm (Asbury Carbons, Asbury, NJ)] to the liquid phantom material before it gelled.

The phantom manufacture procedure was relatively simple. The agarose and potassium sorbate were stirred into the water in a 2‐L beaker, and the mixture was heated in a microwave using 2‐minute cycles of heating and swirling (to ensure that no particles fell out of solution) until the mixture came to a boil. The solution was then boiled for a further minute (until it appeared clear), after which it was cooled slowly to 60°C while being continuously stirred. Once the solution had cooled, a small egg‐shaped mold (approximately 18.4±1 cm^3^) heated to 55°C was filled with some of the solution and kept warm until gelling occurred. The egg‐shaped mold was created by drilling a small hole into the top of a hollow, 2‐piece, plastic Easter egg so that the gel could be introduced. Note that both pre‐heating of the mold and continued warming are required for proper gelling of agarose. The graphite powder was stirred into the remaining solution and a 2‐cm layer of the mixture poured into the plastic phantom (again, pre‐warmed to 55°C) and allowed to gel at 55°C. The egg‐shaped target was then removed from its mold and positioned centrally on the gel layer within the phantom. A further 1‐cm layer of the graphite mixture was then added and gelled to secure the target in position. Once the second layer had gelled, a final layer of graphite mixture was added to provide a total phantom depth of approximately 9.5 cm. The phantom was slowly cooled to room temperature as it gelled and was allowed to sit for several days so that any excess moisture could evaporate.

Several protective measures were added to complete the phantom design. A 3‐mm layer of wax was added to the top of the phantom to protect the gel from the pressure caused by the US probe. To prevent damage to the gel from the water used to provide acoustic coupling between the US probe and the phantom, a layer of plastic film, held in place by a plastic ring, was fitted over the wax. The plastic film is thus easily replaceable if it becomes damaged during routine use of the phantom. As a final step, lines were inscribed on the outside of the phantom to mark the phantom center and to serve as alignment guides for reproducible setup using the lasers in the CT or treatment room.

### B. Phantom testing

#### *B.1 Phantom stability*

Stability at room temperature is a concern with any phantom made of a gel material, because this material has the potential to grow bacteria or fungi or simply to desiccate. To minimize the potential for contamination, potassium sorbate (an effective but nontoxic preservative commonly used in food) was added to the material before it gelled, as a means of preventing growth of bacteria or fungi. As an added precaution, the phantom was scanned by CT on 3 subsequent days 8 months after creation to observe any changes that might have occurred within the phantom. Evaporation of moisture from the gel could also be monitored by CT scanning. In particular, the CT volume of the target was monitored and compared with the volume of the mold. Furthermore, the average monthly volume of the target was determined from the daily ultrasound scans taken during regular clinical use of the phantom. Any problems with phantom desiccation would be exposed by systematic changes in those volumes.

#### *B.2 Tissue‐mimicking properties*

The phantom consists of a gel material that may not be tissue‐mimicking: that is, the speed of sound in the material may not match the speed of sound in tissue. If the gel is not a tissue‐mimicking material, then the position of the centroid and the volume of the target will not be the same on the US images as on the CT images.

We evaluated the tissue‐mimicking properties of the phantom material by acquiring a registered set of CT and US images and by comparing the volumes and positions of the centroid as measured in the two image sets. The CT and US images were obtained in immediate sequence and were implicitly registered using the US‐IGRT system. The manufacturer‐provided software was used to segment the US scan, and the position of the centroid was recorded. The position of the centroid on the CT was determined manually, using the ruler tool available in the Eclipse treatment planning system (Varian Medical Systems, Palo Alto, CA). Both scans were then imported into Eclipse and the US‐to‐CT registration was further examined using a “checkerboard” tool available on that system.

#### *B.3 Reproducibility of positioning measurements*

We carried out tests to determine the reproducibility of target volume segmentation, US scanning, and phantom alignment with the treatment room lasers. Three main tests examined these issues and provided an indication of the usefulness of the phantom as a clinical QA tool.

- The first test involved taking a single US scan of the phantom and segmenting the dataset repeatedly (2 separate tests with 5 trials each), recording the target volume and centroid position each time, as reported by the software. This series of tests was performed by a single physicist (LD).
- The second test involved repeatedly scanning the phantom without moving the phantom between scans. Two such tests were performed, the first with 5 trials, and the second with 10 trials. Once again, the target was segmented using the available software, and the target volume and centroid position were recorded.
- The final test involved repeatedly aligning the phantom with the positioning lasers (using the lines inscribed on the phantom), scanning the phantom using US, and then segmenting the target volume. Before each US scan, the phantom was moved and repositioned using the lasers. This test was conducted on 2 occasions with 5 trials recorded during each session. The mean target volume and centroid position were recorded.

### C. Daily QA tests

Once the stability and reproducibility of the phantom positioning had been determined, the phantom was implemented for daily QA. The checks, performed each day by one of six fully trained radiation therapists, involve lining up the phantom to the positioning lasers in the LINAC bunker, obtaining a single ultrasound scan of the phantom, and using the automated segmentation tools provided with the US‐IGRT system to segment the target volume. The position of the centroid of the segmented target volume is recorded along with the volume of the target. These measurements ensure that the US system is working as intended, that the room and probe calibrations are accurate, and the software is fully functional.

## III. RESULTS

### A. Phantom design and manufacture

**Figure 2:**
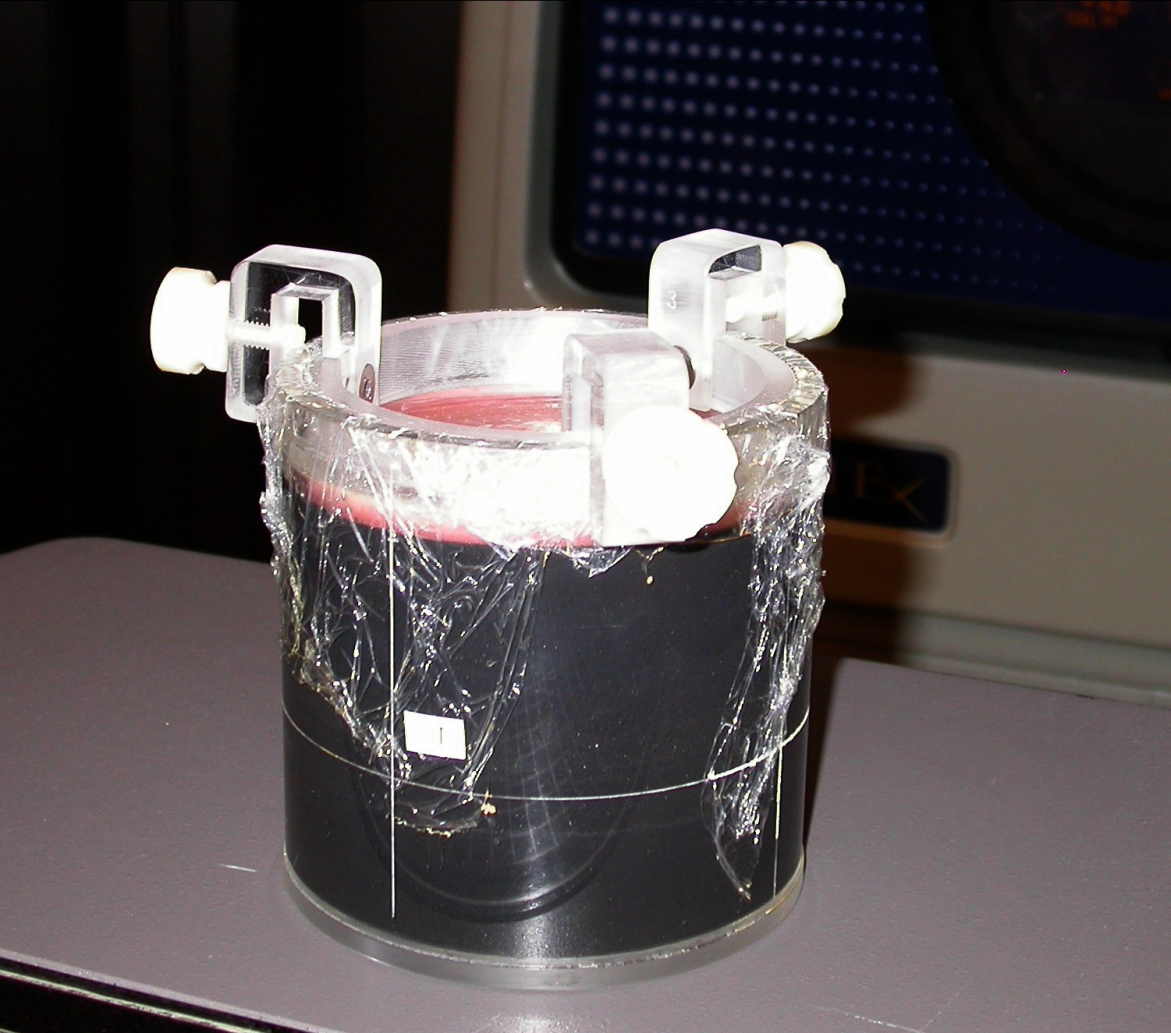
The daily quality assurance phantom positioned for verification of treatment room calibration for the ultrasound image guided radiation therapy system.

**Figure 3:**
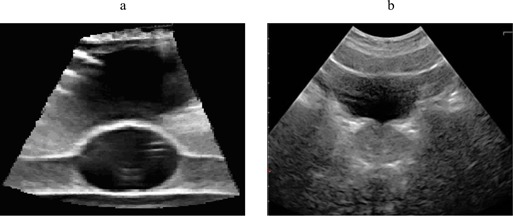
Axial ultrasound image of (a) the daily quality assurance phantom, showing the target and the shadow created by the thin layer of wax, and (b) the clinical patient image.

Fig. 2 shows the daily US QA phantom. The phantom weighs on the order of 1 kg, and it is approximately 13 cm in diameter. Its light weight and small size make it easy to store and use for routine QA, and it is well tolerated by the therapy staff. Fig. 3(a) shows an ultrasound image obtained using the daily QA phantom. In comparison, Fig. 3(b), shows a clinical image of a prostate. The egg‐shaped target volume is clearly visible in the lower part of the image. Also visible are the interfaces that result from the layered construction of the phantom. The interface directly below the target volume mimics the prostate rectum interface in patient images. The shadow above the target volume results from the thin layer of wax applied to the top of the phantom to prevent dents in the gel. This shadowed region is reminiscent of the bladder in US images used for prostate IGRT in patients. The similarities exhibited by the phantom images and the patient images are true clinical benefits of this phantom system, because they allow radiation therapists to view and segment images with confidence.

### B. Phantom testing

#### *B. 1 Phantom stability*

**Figure 4:**
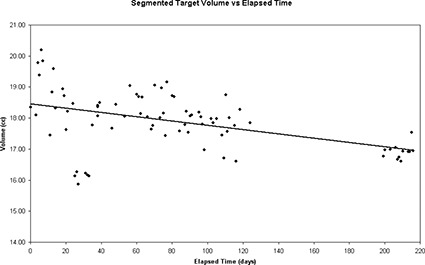
Target volume, as measured daily using ultrasound, versus the elapsed time since the creation of the phantom. Because no patients were on treatment between day 120 and day 200, the daily quality assurance (QA) of the ultrasound system was not performed, producing a gap in the data points. The data points collected between day 25 and day 33 are the result of an untrained user performing the QA procedure.

The stability of the phantom over time was investigated by recording the volume of the segmented egg‐shaped target as seen on the daily US scans several times per month for the first 220 days after the phantom was constructed. Fig. 4 shows the recorded volume since phantom creation. Between day 25 and day 33, the cluster of points that fall below the other data were obtained by a user who had not been instructed in the use of the phantom. As a result, the images taken over these days were of lower quality and were not properly segmented. An observed slight linear decrease in target volume (*V*) is given by the equation

(1) $$V=-0.006893\;cm^{3}/\;day\;\;\times \;t+18.46\;cm^{3}\;,$$

where *t* is the elapsed time in days since phantom creation. The coefficient of determination is 0.22. The downward slope indicates that, during the course of a month, the volume of the target will decrease by approximately 0.2 cm^3^.

The volume of the target was also measured using 3 sets of CT images taken on succeeding days 8 months after the phantom was created. The CT‐measured target volume was compared to the volume of the mold used to create the target. The average target volume was found to be 18.3±0.7 cm^3^, but the volume of the mold is 18.4±1.0 cm^3^. Notably, the intercept value of the target volume, as shown in equation 1, falls within the error of the volume of the mold used to create the target volume. These results also indicate that, although the US‐determined volume appears to be decreasing, the CT‐determined target volume still agrees, within error, to the volume of the mold.

#### *B.2 Tissue‐mimicking properties*

**Figure 5:**
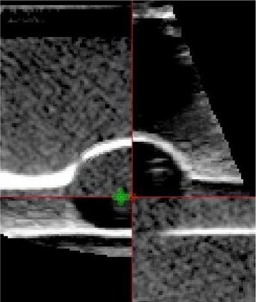
Merged computed tomography (CT) and ultrasound (US) image of the daily quality assurance phantom. The upper left and lower right quadrants are CT images; the other quadrants are from the US system. Note the boundary between the target and the background that is visible in both images.

With the phantom positioned in a single location, CT and US images were obtained and implicitly registered using the US‐IGRT system. Fig. 5 shows a “checkerboard” of the merged images from the Eclipse system. The target is quite well aligned between the two sets of images. The anterior and posterior boundaries of the target volume are slightly higher on the US image than on the CT image; however, the lateral boundaries are aligned. This result suggests that the speed of sound in the phantom material is slightly higher than that assumed for soft tissues by the US imaging system. Table 1 shows the position of the target centroid as recorded for both the CT and US datasets. Using the anterior–posterior centroid positions for CT and US from Table 1, and the dimensions of the phantom (64.0±0.5 mm from the surface of the phantom to the DICOM centre of the CT image), the speed of sound in the phantom material was approximated at 1552±67 m/s.

**Table 1:** Comparison of centroid position of egg‐shaped target as measured on computed tomography (CT) and ultrasound (US) images of the phantom

	Centroid position (mm)	
Direction	CT (±0.1)	US (±0.05)
Superior–inferior	1.7	1.68
Left–right	2.1	1.95
Anterior–posterior	2.6	3.08

#### *B.3 Reproducibility of Positioning Measurements*

For the phantom to be useful for daily US QA, it must perform reproducibly. We performed three tests to determine how reproducibly the daily QA phantom could be aligned with the lasers and scanned using US, and how reproducibly the target volume could be segmented. These tests were first conducted soon after the phantom was created; they were repeated 4 months later. Tables 2 – 4 show the mean and standard deviation of the centroid position and volume for the tests of repeated target segmentation, US scanning, and phantom setup.

**Table 2:** Centroid position and volume of the egg‐shaped target as determined by repeat segmentation of a single ultrasound image of the daily quality assurance phantom

	Month 1	Month 4
Volume (cm^3^)	18.55±0.05	18.29±0.02
Superior–inferior position (mm)	1.87±0.08	1.33±0.01
Left–right position (mm)	1.05±0.06	2.97±0.03
Anterior–posterior position (mm)	1.30±0.01	1.79±0.01

**Table 3:** Centroid position and volume of the egg‐shaped target as determined by repeat ultrasound scanning and segmentation of the daily quality assurance phantom

	Month 1	Month 4
Volume (cm^3^)	19.0±0.2	18.8±0.2
Superior–inferior position (mm)	1.3±0.4	1.5±0.3
Left–right position (mm)	1.3±0.1	2.6±0.3
Anterior–posterior position (mm)	0.97±0.07	1.65±0.05

**Table 4:** Centroid position and volume of the egg‐shaped target as determined by repeated setup, ultrasound imaging, and segmentation of the daily quality assurance phantom

	Month 1	Month 4
Volume (cm^3^)	18.8±0.3	18.3±0.3
Superior–inferior position (mm)	0.9±0.7	1.3±0.8
Left–right position (mm)	1.1±0.2	2.8±0.3
Anterior–posterior position (mm)	1.0±0.5	1.6±0.3

### C. Daily QA testing

**Figure 6:**
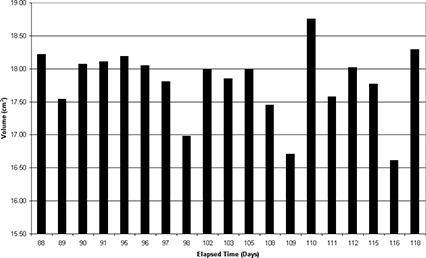
Segmented volume measured using ultrasound from daily quality assurance versus elapsed time for a 1‐month period following the last upgrade.

**Figure 7:**
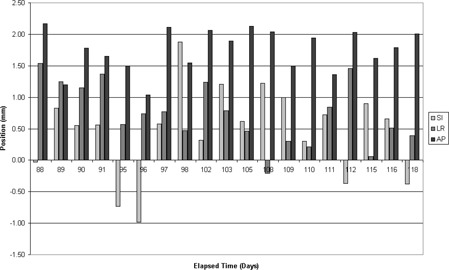
Centroid position of the target volume as found by the daily quality assurance of the phantom, for a 1‐month period following the last upgrade.

The volume of the target and the position of the centroid were recorded during the daily QA testing performed by the radiation therapists over a period of 220 days. Figs. 6 and 7 respectively show a 1‐month subset of these target volume and centroid position measurements, obtained immediately following an upgrade and calibration of the US‐IGRT system. To analyze the typical variation in observed readings, 1 month was used because the US‐recorded target volume has been shown to decrease slowly over time (Fig. 4) and a monthly recheck of baseline values has been recommended. Over the 1‐month period shown in Figs. 6 and 7, the recorded target volume fell within ±1 cm^3^ and the target centroid positions fell within ±1.5 mm of the mean position in each of the three dimensions.

## IV. DISCUSSION

The daily QA phantom constructed at our clinic met all the design goals for a phantom to perform simple US‐IGRT QA on a regular basis. The phantom is approximately one tenth the weight of the manufacturer‐provided phantom and thus offers substantial advantages. It is easy to lift and position on the treatment couch and is therefore less likely to be dropped. The structures in the phantom are easy to segment because the target is highly visible against the background. Finally, the phantom has proved to be highly robust: in the 9 months since it was created, it has not decayed, and desiccation has been minimal. Combined observations over time of a stable CT‐measured target volume and a slightly lesser US‐measured target volume suggests that a minor amount of desiccation is occurring. The desiccation of the gel is large enough to produce small changes in the speed of sound in the gel, but insufficient to alter the volume of the target as measured with CT. The decrease in the US‐measured target volume indicates that the speed of ultrasound is increasing with time, a characteristic observed in other phantom materials.^(^ ^8^ ^,^ ^9^ ^)^ Because of the small changes observed, our recommendation is that, each month during routine QA and calibration of the US‐IGRT system, the daily QA phantom be examined and, if required, the baseline values of the target position be adjusted. Following the monthly check of system calibration using the commercial calibration phantom, the daily QA phantom should be imaged with the US‐IGRT system and a new target volume and a new centroid position computed. These values provide a new baseline to which the daily QA results can be compared.

The structures within an US image of the phantom were found to match very well with a registered CT image. A slight discrepancy (<1 mm) in target location between US and CT is observed in the anterior–posterior direction. This result indicates that the speed of sound in the gel does not exactly match that of soft tissue (to which the US machine is calibrated), and thus the phantom material is not exactly tissue‐mimicking. However, because the phantom is used for relative checks of the target centroid and volume against baseline values, and not for absolute calibration, this small difference is quite acceptable. An additional design benefit of this phantom is the striking similarity between the US phantom and the patient images that are typically acquired for prostate US‐IGRT. This similarity to the clinical images allows the radiation therapists to view and segment the phantom images with confidence.

Tests of the reproducibility of QA measurements made using the phantom indicate that this tool is highly reliable for daily QA of the localization ability of an US‐IGRT system. The results of the segmentation test (Table 2) show that the position of the centroid of the target volume is highly reproducible upon multiple segmentations. For all measurements, the standard deviation is <0.1 mm. The standard deviations are generally larger for measurements made in month 1 than they are when the test is repeated in month 4. This improvement in precision likely occurred because of increased user experience over time. The main source of variability is the US imaging and not the positioning of the phantom or the segmentation of the images. Notably, the mean volume and position for month 1 and month 4 do not necessarily agree, because the US‐IGRT system underwent an upgrade and recalibration during that time. The results for the scanning test of the phantom (Table 3) also show excellent reproducibility, with <0.4 mm for all standard deviations in target position.

The final test of phantom reliability shows the setup accuracy of the phantom (Table 4). Again, the reproducibility is very good. The largest standard deviation in the position of the centroid occurs in the superior–inferior direction (0.8 mm as compared with approximately 0.3 mm), as was the case with the scanning test. The larger variation in the superior–inferior direction likely occurs as the result of a dependence of the reconstruction software on the direction of scan. At our clinic, this dependence was observed early on, because of the position of the infrared tracking camera. It highlighted a problem with the reconstruction software that was corrected in subsequent versions. In summary, these results indicate that the daily QA phantom can reproducibly measure target location within a precision of <1 mm when measurements are performed by a single user.

Results for clinical use of the daily QA phantom were also very good. As shown in Figs. 6 and 7, the level of precision achievable with this system remains good when it is used in a typical clinical environment with multiple radiation therapists making daily measurements. The system allows clinically reasonable tolerances of ±2 mm on the target centroid position and ±1 cm^3^ on the target volume to be placed on daily QA measurements for the US‐IGRT system. The daily QA using the phantom detected no occurrences of the system being out of tolerance, but it did find occurrences of the system being interlocked and requiring reboot. That finding is a reflection of our well‐functioning weekly and monthly QA programs and the stability of the US‐IGRT system over this time frame.

## V. CONCLUSIONS

The phantom described in this paper can be used for daily reliability checks of an US‐IGRT system. The phantom provides reproducible measures of target volume and location and can be used clinically with tolerances of ±1 cm^3^ on the segmented volume and ±2 mm on the centroid position. The phantom has many features that make its use desirable. Among those features are its small size and light weight, and the similarity of its US images to images obtained for prostate IGRT. Furthermore, the phantom material has not significantly degraded over a period of 9 months, and the phantom is stable at room temperature. For daily QA, this phantom is a good alternative to the calibration phantom supplied by the manufacturer; we recommend that the supplied calibration phantom be reserved for less frequent (for example, monthly) QA tests and for system calibration.
